# Physio-Biochemical and Transcriptomic Features of Arbuscular Mycorrhizal Fungi Relieving Cadmium Stress in Wheat

**DOI:** 10.3390/antiox11122390

**Published:** 2022-12-01

**Authors:** Hua Li, Hongxia Wang, Jianan Zhao, Lele Zhang, Yang Li, Huijuan Wang, Huixin Teng, Zuli Yuan, Zhiliang Yuan

**Affiliations:** College of Life Science, Henan Agricultural University, Zhengzhou 450002, China

**Keywords:** arbuscular mycorrhizal fungi, cadmium, GSH metabolism, methylglyoxal, transcriptomes

## Abstract

Arbuscular mycorrhizal fungi (AMF) can improve plant cadmium (Cd) tolerance, but the tolerance mechanism in wheat is not fully understood. This study aimed to examine the physiological properties and transcriptome changes in wheat inoculated with or without *Glomus mosseae* (GM) under Cd stress (0, 5, and 10 mg·kg^−1^ CdCl_2_) to understand its role in wheat Cd tolerance. The results showed that the Cd content in shoots decreased while the Cd accumulation in roots increased under AMF symbiosis compared to the non-inoculation group and that AMF significantly promoted the growth of wheat seedlings and reduced Cd-induced oxidative damage. This alleviative effect of AMF on wheat under Cd stress was mainly attributed to the fact that AMF accelerated the ascorbate-glutathione (AsA-GSH) cycle, promoted the production of GSH and metallothionein (MTs), improved the degradation of methylglyoxal (MG), and induced GRSP (glomalin-related soil protein) secretion. Furthermore, a comparative analysis of the transcriptomes of the symbiotic group and the non-symbiotic group revealed multiple differentially expressed genes (DEGs) in the ‘metal ion transport’, ‘glutathione metabolism’, ‘cysteine and methionine metabolism’, and ‘plant hormone signal transduction’ terms. The expression changes of these DEGs were basically consistent with the changes in physio-biochemical characteristics. Overall, AMF alleviated Cd stress in wheat mainly by promoting immobilization and sequestration of Cd, reducing ROS production and accelerating their scavenging, in which the rapid metabolism of GSH may play an important role.

## 1. Introduction

Continuous industrial waste discharge and pesticide and fertilizer application have increased soil pollution with heavy metals (HM) [1]. According to a previous survey, the HM content in 16.1% of the soil samples in China exceeds the standard levels set by the Ministry of Environmental Protection (MEP), with cadmium (Cd) being the highest pollutant, accounting for 7% [2]. Cd-contaminated soil is the main source of Cd in plants, since it is easily absorbed by plant roots from the soil [3]. Furthermore, Cd toxicity inhibits plant growth and development and threatens human health through the food chain cycle [4]. Therefore, reducing Cd toxicity and enhancing its tolerance in plants has become a major focus for sustainable agriculture and human health.

Upon exposure to excess Cd, plant cells produce numerous reactive oxygen species (ROS), which causes oxidative stress, protein denaturation, lipid peroxidation, and nucleotide degradation, ultimately leading to cell damage and even death [5]. Thus, plants have evolved two strategies to deal with the Cd-induced oxidative damage: tolerance and avoidance [6]. Plant tolerance to Cd stress mainly refers to ROS scavenging through non-enzymatic and enzymatic antioxidative defense systems, thereby reducing the oxidative damage to cells [7,8]. Non-enzymatic antioxidants include glutathione (GSH), ascorbate (AsA), carotenes, and α-tocopherol. Enzymatic antioxidants mainly comprise superoxide dismutase (SOD), catalase (CAT), peroxidase (POD), and the antioxidant enzymes involved in the AsA-GSH cycle, such as ascorbate peroxidase (APX), glutathione peroxidase (GPX), monodehydroascorbate reductase (MDHAR), dihydroascorbate reductase (DHAR), and glutathione reductase (GR) [9]. Certainly, these antioxidant resistance mechanisms against Cd toxicity are also applicable in wheat [10]. For the avoidance strategy, plants directly reduce Cd content by decreasing its absorption from soil or inhibiting Cd transport from roots to shoots. Plants also combine free Cd ions with phytochelatins (PC) and metallothionein (MTs) to form complexes. Poly (γ-glutamyl-cysteinyl)n glycine, (PC-Gly, where *n* = 2–11) is a ubiquitous PC in angiosperms; additionally, hydroxymethyl-phytochelatin and poly (γ-glutamyl-cysteinyl)n serine, (PC-Ser) are also found in *Poaceae* (including wheat) [11]. MTs are low molecular weight proteins (<10 KDa) that are rich in highly conservative CC, CXC, and CXXC motifs, which can bind with different metal ions [12]. Cd^2+^ can combine with PCs and MTs to form chelates (Cd^2+^-PCs and Cd^2+^-MTs), which are then transported into vacuoles for sequestration and detoxification [13,14]. Additionally, organic acids (such as citric acid, oxalic acid, malic acid, salicylic acid, phytic acid, lipoic acid, and phenolic acids) secreted by plant rhizospheres can also chelate HMs’ cations through their carboxyl group (COOH) to form complexes, thus reducing the effective concentration of HM elements [15].

Arbuscular mycorrhizal fungus (AMF) is a symbiotic endomycorrhizal fungus widely distributed on more than 90% of terrestrial plant species [16]. The extraradical and intraradical hyphae and arbuscules of AMF facilitate water and nutrient supply to host plants, thereby improving growth and yield [17,18,19]. AMF also enhances plant tolerance to HMs by modulating various stress-alleviating physiological processes in host plants [20,21,22]. The extraradical hyphae of AMF could secrete glomalin (measured as glomalin-related soil protein (GRSP) in soils), which is hydrophobic, thermally stable, and recalcitrant (resistant to decomposition). GRSP can combine with carbon, nitrogen, phosphorus and some metal ions [23], so GRSP plays an important role in improving plant growth and plant tolerance to HMs [24]. The combination of GRSP and HMs reduces HMs’ biological activity by changing metal speciation in the rhizosphere, thus mitigating HMs’ toxicity to host plants [25,26,27,28]. Additionally, several reports have shown that the intraradical and extraradical mycelia surfaces of AMF could adsorb HMs, thus reducing the HMs’ uptake in the host plants [29,30,31]. Furthermore, AMF could alleviate HMs’ stress by regulating the biosynthesis of some molecules (such as antioxidant enzymes, chelates, and glomalin) involved in the plant defense systems and related gene expression [22,32].

Wheat (*Triticum aestivum* L.), the main food crop worldwide, can accumulate more Cd via its roots and translocate it to the aerial parts, including the wheat grains, which surpasses the standard Cd levels considered safe in grains [33,34]. High Cd levels in wheat grains seriously threaten human health [35]. Additionally, studies have reported positive correlations between Cd levels in soil and wheat grains [36], and this necessitates an urgent development of in situ technologies to remediate Cd-contaminated farmlands. AMF has a great potential for ameliorating heavy metals in plants and is also an inexpensive and eco-friendly bioremediation tool for heavy metal-polluted soils [37]. Although there are several studies on Cd-mycorriza-wheat, there is no study that deeply discusses the mechanism of AMF in relieving Cd toxicity in wheat. Therefore, this study aimed to interpret the mechanisms of AMF alleviating Cd toxicity in wheat by evaluating the oxidative damage, AsA-GSH cycle, methylglyoxal (MG) metabolism, glomalin production, Cd chelation, and transcriptome characteristics associated with Cd stress. Recently, six families of AMF were found in the wheat rhizosphere through DNA sequencing, including *Archaeosporaceae*, *Claroideoglomeraceae*, *Diversisporaceae*, *Gigasporaceae*, *Glomeraceae*, and *Paraglomeraceae* [38], in which *Glomeraceae* has been widely proven to regulate wheat growth and development and abiotic stress; therefore, *Glomus mosseae* (GM, belongs to *Glomeraceae*) was used for symbiosis with wheat in this study.

## 2. Materials and Methods

### 2.1. Plant Growth and Treatment

Seeds of the wheat variety Bai Nong 207 (BN207) were sterilized with 5% H_2_O_2_ for 5 min. For the non-inoculation treatment group, the seeds were sown into plastic pots containing 120 g of sterilized soil (vermiculite:soil; 1:3, *w*/*w*). Meanwhile, pots containing 115 g sterilized soil inoculated with 5 g arbuscular mycorrhizal fungus (*Glomus mosseae*; GM) inoculum were used for sowing the seeds for the inoculated treatment group. The inoculum was provided by BGC strain bank of the Institute of Plant Nutrition and Resources, Beijing Academy of Agriculture and Forestry Sciences. All pots were supplemented with a half-strength Hoagland nutrient solution. After three days of culture, both the non-inoculation and the inoculation plants were divided into three groups, which were treated with 0, 5, and 10 mg·kg^−1^ CdCl_2_, one each. Each treatment contained at least 5 pots and each pot planted with 20 seedlings. Thereafter, all groups were supplemented with a half-strength Hoagland nutrient solution to maintain soil moisture at 80%. The seedlings were cultured under 16 h light/8 h dark, at temperatures of 16–25 °C, illumination of 0–100 μmol·m^−2^·s^−1^, and 80% humidity. The leaves, roots, and culture soil were collected after 15 days of Cd treatment. All samples were collected and immediately frozen in liquid nitrogen, and then stored at −80 °C.

### 2.2. Measurement of Mycorrhizal Colonization

The harvested roots were rinsed with deionized water and cut into segments about 1 cm long, and then the roots were stained with ink-vinegar solution according to the method of Vierheilig et al. [39]. Stained roots were randomly selected to assess root colonization by the gridline intersection method [40], and microscopy BX51 (OLYMPUS, Tokyo, Japan) was used to visualize mycorrhizal colonization.

### 2.3. Determination of Hydrogen Peroxide, Superoxide Radicals, and Malondialdehyde Content

Previously published methods were used to determine the hydrogen peroxide (H_2_O_2_) [41], superoxide radicals (O_2_^•−^) [42], and malondialdehyde (MDA) content [43] of the wheat leaves. For H_2_O_2_ determination, the samples (0.1 g) were ground in liquid nitrogen and transferred into 0.1% trichloroacetic acid (4 mL), centrifuged at 4 °C, 12,000× *g* for 10 min, and then 1 mL supernatant was added to reaction solution (containing 0.5 mL 10 mM PBS (pH 7.0) and 1 mL 1 M KI) to measure the absorbance at 390 nm. For O_2_^•−^ determination, 0.1 g samples were transferred to 3 mL 65 mM PBS (pH 7.8) after being ground in liquid nitrogen, centrifuged at 4 °C, 5000× *g* for 10 min, and then 0.75 mL supernatant was added to 0.675 mL 50 mM PBS (pH 7.8) and 0.07 mL 10 mM hydroxylamine hydrochloride, followed by a water bath at 25 °C for 20 min after 5 min of ultrasound; afterward, an equal volume of trichloromethane was added for extraction, and the upper pink water phase was used to measure the absorbance at 530 nm. For MDA determination, 0.1 g samples were ground in liquid nitrogen and transferred into 10 mL 10% trichloroacetic acid, centrifuged at 4 °C, 4000× *g* for 10 min, then the mixed solution (2 mL supernatant and 2 mL 0.6% TBA solution) was boiled for 15 min, centrifuged at 4 °C, 4000× *g* for 15 min after cooling, and finally, the absorbance of the supernatant at 450 nm, 532 nm and 600 nm was determined.

### 2.4. Determination of Glutathione and Ascorbate Redox Status

Wheat leaves (0.1 g) were ground in 5% (*w*/*v*) TCA (trichloroacetic acid) and centrifuged at a speed of 12,000 rpm for 10 min at 4 °C. The supernatant was used to determine the quantities of GSH, GSSG, AsA, and DHA. The glutathione redox status was determined using the method by Brehe and Burch [44], while the method by Gossett [45] was used for that of ascorbate. In detail, add 2.0 mL supernatant to the reaction solution containing 0.5 mL 10 mM dithiothreitol (DDT), 0.5 mL 0.5% N-ethyl maleimide (NEM), 0.5 mL 0.4% H_3_PO_4_ (dissolved in ethanol), 1.0 mL 4% bipyridine and 0.5 mL 0.3% FeCl_3_, then put the reaction system into a water bath at 30 ℃ for 40 min and measure the absorbance at 534 nm to determine the total ASA (T-AsA) content. To determine the content of reduced AsA (AsA), 1.0 mL absolute ethanol was used instead of DDT and NEM, and other operations were the same as the determination method of T-AsA. The content of dehydroascorbic acid (DHA) is the difference between T-AsA and AsA. For the determination of total glutathione content (T-GSH), mix 0.2 mL supernatant, 2 mL 0.2 M PBS (pH 7.5), 0.3 mL 2 mM NADPH, 0.3 mL 1.5 U GR, and 0.2 mL 6 mM DTNB, and then measure its absorbance at 412 nm. GSH measurement was made by detecting the absorbance of the mixed solution (0.2 mL supernatant, 2.6 mL 0.1 M PBS (pH 7.7) and 0.2 mL 1.2 mM DTNB) at 412 nm. The GSSG content was calculated as T-GSH minus GSH.

### 2.5. Measurement of Ascorbate-Glutathione Cycle-Related Enzymes and Glutathione S-Transferase

The leaf samples (0.1 g) were ground in liquid nitrogen and mixed with 5 mL 50 mmol·L^−1^ phosphate buffer (pH 7.6) and the samples were centrifuged at a speed of 12,000× *g* for 20 min at 4 °C. The obtained supernatant was used to measure the MDHAR [46] and DHAR [47] activities. Specifically, add 0.5 mL of supernatant into the reaction solution (2.5 mL 50 mM phosphate buffer (pH 7.6), 2.5 mL 7.5 mM AsA, 0.5 mL 25 mM NADPH, and 0.5 mL 1.5 U ascorbic acid oxidase), and then test the MDHAR activity at 340 nm after mixing. Add 0.1 mL of supernatant into the reaction solution (2.0 mL 50 mM phosphate buffer (pH 7.0), 0.5 mL 20 mM GSH, and 0.5 mL 2 mM DHA), and then test the DHAR activity at 265 nm after mixing. For APX, GPX, GR, and glutathione S-transferase (GST) activities, their tests were conducted using kits of QS1304, MS1202, MS1111, and MS1204 respectively, which were purchased from Solarbio, Beijing, China.

### 2.6. Measurement of Cysteine, Metallothionein, and Phytochelatins Contents and the Activities of Phytochelatin and Glutathione Synthase

Leaf samples (0.1 g) were ground in liquid nitrogen, mixed with 0.9 mL of 10 mmol·L^−1^ phosphate buffer (pH 7.4), and centrifuged at a speed of 4000× *g* for 15 min at 4 °C. The supernatant was used to measure cysteine, metallothionein (MT), and phytochelatin (PC) contents and the activity of glutathione synthase (GS); their detection was conducted using ELISA kits (Mlbio, Shanghai, China) of YJ096521, YJ022551, YJ029652 and YJ041369.

### 2.7. Determination of Cadmium Content in the Shoots and Roots

Cd content of wheat shoots and roots was determined using a previously published method [48]. Briefly, wheat leaves and roots were dried at 80 °C, pulverized, and passed through a 40-mesh screen. Thereafter, 0.25 g of the samples was placed in a Teflon tube containing 3 mL of HNO_3_ and 2 drops of H_2_O_2_ for digestion at 110 °C for 30 min. After cooling the digest to room temperature, the extract was filtered through a 0.2 μm cellulose acetate membrane, and 1 mL of the filtrate was transferred into a volumetric flask and filled up to 10 mL with 3% sodium nitrate. The Cd content was then determined using an inductively coupled plasma mass spectrometer (ICP-MS, X Series 2).

### 2.8. Determination of Superoxide Dismutase, Catalase, and Peroxidase Activities

We ground 0.2 g of wheat leaves in 1.5 mL of pre-cooled 50 mM PBS and centrifuged the mixture at a speed of 15,000 rpm for 20 min at 4 °C. The obtained supernatant was then used to determine the enzyme activities of SOD [49], CAT [50], and POD [51]. For SOD activity determination, 2.3 mL 50 mM PBS (pH 7.0), 0.2 mL 95 mM methionine, 0.1 mL 0.3 mM EDTA · 2Na, 0.05 mL supernatant, 0.2 mL 1.125 mM EDTA · 2Na and 0.2 mL 60 μM riboflavin were added into the reaction system in sequence, then the reaction solution was placed under the lamp (4000 lx, 25 °C) for 10 min, and the absorbance at 560 nm was measured. For CAT activity determination, 2.6 mL 50 mM PBS (pH 7.8), 0.1 mL supernatant, and 0.3 mL 100 mM H_2_O_2_ were successively added to the reaction system, then we quickly measured the absorbance at 240 nm and took reads every 20 s for 3 min. The enzyme activity of SOD is defined as the change of H_2_O_2_ decomposed per minute per gram of protein. For POD activity determination, 4 mL 50 mM PBS (pH 5.5), 1 mL 30 mM guaiacol, 1 mL supernatant, and 1 mL 0.6% H_2_O_2_ were added into the reaction system in turn, then we quickly measured the absorbance at 470 nm and took reads every 20 s for 3 min. The enzyme activity of POD was defined as the change of A_470_ per minute per gram of protein.

### 2.9. Determination of Methylglyoxal Content and the Activities of Glyoxalase I and II

A previously published method [52] was used for methylglyoxal (MG) content determination. Briefly, 0.5 g wheat leaves were ground in 3 mL of pre-cooled 0.5 M perchloric acid, and the mixture was centrifuged at a speed of 11,000× *g* for 10 min at 4 °C. The supernatant was placed in sterile centrifuge tubes containing 10 mg·mL^−1^ activated carbon, decolorized at room temperature for 15 min, and then centrifuged at a speed of 11,000× *g* for 10 min at 4 °C. The pH of the obtained supernatant was adjusted to 7.0 with saturated potassium carbonate solution and kept at room temperature for 15 min. Afterward, 650 μL of the supernatant was added into a mixture of 250 μL of 7.2 mM phenylenediamine and 100 μL of 5 M perchloric acid. After 25 min of the reaction, the absorbance was read at 335 nm. At the same time, MG with different concentrations was used to replace the supernatant, and the above determination process was repeated to obtain the standard curve. Finally, the MG content in samples was calculated based on the generated standard curve. Moreover, glyoxalase I and II activities were measured using ELISA kits (MEIMIAN, Yancheng, China) of MM-63488O1 and MM-35918O1, respectively.

### 2.10. Determination of the Glomalin Related Soil Protein Content in Soil and the Cadmium Content in Glomalin Related Soil Protein

A previous method [53] was adopted to determine the GRSP content of rhizosphere soil. Briefly, 0.75 g air-dried soil was added into 6 mL of 20 mM sodium citrate buffer (pH 7.0), mixed ultrasonically, and sterilized at 121 °C for 30 min. The mixture was then centrifuged at 10,000× *g* for 20 min, and the obtained supernatant was used to measure the GPRS content using the Bradford method [54]. For the Cd content in the GRSP, 2 M HCl was slowly added to the supernatant until pH 2.5 was obtained, and the acidified supernatant was centrifuged at 8000× *g* for 10 min, according to a slightly modified method by Wu et al. [26]. The obtained residue was redissolved in 0.1 M sodium borate (pH 9.0), dialyzed against ultrapure water, and lyophilized. The lyophilized GRSP was mixed with 4 mL of HNO_3_ and 2 mL of H_2_O_2_ and digested in a microwave digestion system for 5 min, after which the temperature was slowly raised to 110 °C and maintained for 30 min. After cooling the Teflon tube to room temperature, the extract was filtered through a 0.2 μm cellulose acetate membrane, and 1 mL of the filtrate was topped up to 10 mL with 3% sodium nitrate. The solution was then used for Cd content determination via an inductively coupled plasma mass spectrometer (ICP-MS, X Series 2).

### 2.11. Transcriptome Analysis

The total RNA was extracted from the wheat leaves using the TRIzol reagents (Invitrogen, Carlsbad, California, USA). Thereafter, 5 μg of the total RNA was used to construct cDNA libraries using the KC-DigitalTM RNA kit, and the constructed cDNA libraries were sequenced by Novaseq 6000 sequencer (Illumina) PE150 at Wuhan Kangce Technology Co., Ltd. In this study, 12 libraries were constructed: four treatments (CK, GM, Cd10, and GM+Cd10) × three replicates. We then filtered the raw sequencing data (raw data) using Trimmomatic software (version 0.36) to remove low-quality reads and those containing adapters and eliminated duplication biases introduced during library preparation and sequencing using the UMI-software (Wuhan Seqhealth Co., version 1.0). Finally, about 50 million reads were obtained from each library, and all the RNA-Seq clean data care available at the Sequence Read Archive (SRA) (https://submit.ncbi.nlm.nih.gov/subs/sra/ accessed on 1 November 2022) under the accession number PRJNA854774.

The wheat genome sequence (IWGSC RefSeq v1.0) served as the reference genome. The STAR software (version 2.5.3a) was used to compare the clean reads with the reference genome to determine gene positions and sequence characteristics specific to the sequenced sample. The differential genes (DEG) between groups were screened using the edgeR software package (version 3.12.1), and the genes were regarded as DEGs if |log FC| > 1 and *p*-Value < 0.05. The obtained DEGs were subjected to GO and KEGG analyses using KOBAS software (version 2.1.1).

### 2.12. qRT-PCR Analysis

Leaves were ground in liquid nitrogen, total RNA was isolated by the TRIzol reagent, and reverse transcription reactions were conducted by using PrimeScript^®^ RT Reagent Kit with gDNA Eraser (Takara, Kyoto, Japan). qRT-PCR experiments were performed on an ABI StepOne real-time PCR instrument by using Novozymes AceQ^®^ qPCR SYBR Green Master MIX kit. Actin was used as the internal reference gene. The relative expression levels of detected genes were calculated by the 2^−ΔΔCt^ method. The primer sequences used in this study are shown in Supplementary Materials S1.

### 2.13. Statistical Analyses

All the data obtained were analyzed using Data Processing System (DPS v 14. 10) software. One-way ANOVA was performed for comparing significant differences among treatments, and *p* < 0.05 served as the significance threshold.

## 3. Results

### 3.1. Mycorrhizal Colonization

No mycorrhizal colonization was found in non-inoculation group, but mycorrhizal colonization was observed in symbiotic inoculation. The root colonization rate was 43.2%, 38.7%, and 36.5% under 0, 5, and 10 mg·kg^−1^ CdCl_2_ treatment, respectively. The addition of Cd slightly reduced the mycorrhizal colonization rate (Supplementary Materials S1).

### 3.2. Effects of AMF on Wheat Growth and Cd Accumulation under Cd Stress

**Figure 1:**
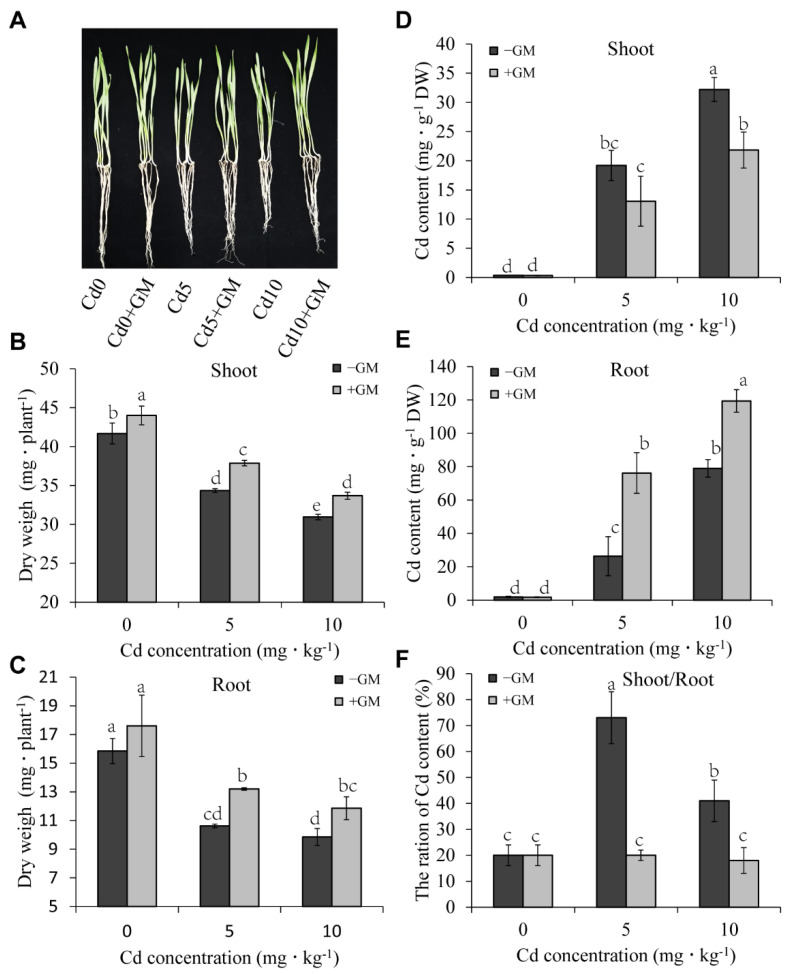
Effects of AMF on wheat growth and Cd content under Cd stress. The changes of phenotype (**A**), dry weight of shoots (**B**) and roots (**C**), Cd content in shoots (**D**) and roots (**E**), and its ratio in shoots/roots (**F**) under Cd stress. Values are the means ± standard deviation (SD) (*n* = 3). Different letters in the same column indicate statistically significant differences (*p* < 0.05).

Inoculation of wheat with AMF enhanced the growth of wheat plants under Cd-stress conditions (Figure 1A). AMF increased significantly the dry weight of wheat shoots by 10.3% and 8.8% (Figure 1B) and enhanced the dry weight of wheat roots by 24.2% and 20.4% under 5 and 10 mg·kg^−1^ CdCl_2_ treatments, respectively (Figure 1C). Additionally, AMF significantly reduced Cd content in the leaves but increased Cd accumulation in the roots. Under 5 and 10 mg·kg^−1^ CdCl_2_ treatments, the Cd content was reduced by 21.8% and 32.2% in the leaves of the inoculation groups compared to non-inoculation groups, respectively (Figure 1D); however, the Cd content increased by 189.4% and 51.1% in the roots of inoculation groups compared to non-inoculation groups, respectively (Figure 1E). Furthermore, the AMF inhibited the Cd transport rate (shoot/root) from the roots to the shoots by 72.1% and 55.1% under 5 and 10 mg·kg^−1^ CdCl_2_ treatments, respectively (Figure 1F).

### 3.3. Effects of AMF on the GRSP Content of Rhizosphere Soil and Cd Content Bound by the GRSP under Cd Stress

**Figure 2:**
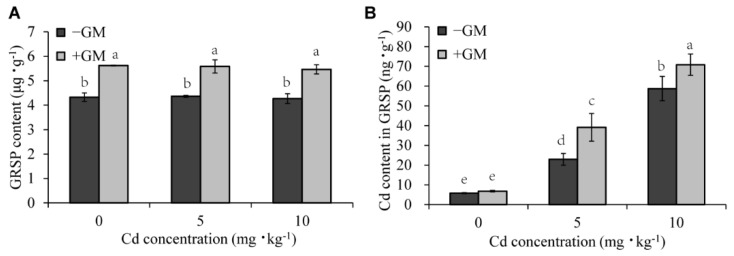
Changes in the content of GRSP secreted by AMF (**A**) and the content of Cd in GRSP (**B**) under different Cd concentrations. Values are the means ± standard deviation (SD) (*n* = 3). Different letters in the same column indicate statistically significant differences (*p* < 0.05).

Previous studies have shown that AMF can adsorb GRSP-integrated heavy metals using their extraradical hyphae, inhibiting the transport of heavy metals from roots to shoots, thereby reducing their damage [55]. In this study, higher GRSP accumulation was observed in the rhizosphere soil of the inoculation group compared to the non-inoculation group, and this accumulation had nothing to do with whether there was Cd treatment (Figure 2A). The GRSP content in the rhizosphere soil of the inoculation group was 30.0%, 28.1%, and 28.1% higher than that of the non-inoculation group under 0, 5, and 10 mg·kg^−1^ CdCl_2_ treatment, respectively. The results of Cd content in GRSP showed that with the increase in Cd concentration, the Cd content bound by GRSP increased, and AMF symbiosis significantly enhanced this effect. That is, the Cd content in GRSP was higher in the inoculation group than in the non-inoculation group under Cd stress (Figure 2B).

### 3.4. AMF Reduces Cd-Induced Oxidative Damage in Wheat Leaves

**Figure 3:**
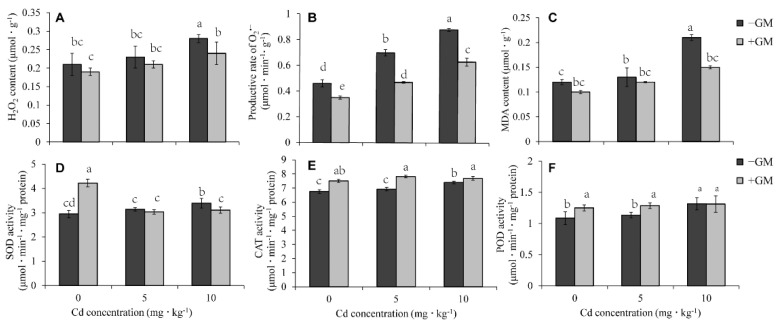
Effects of AMF on H_2_O_2_, O_2_^•−^, MDA content (**A**–**C**) and SOD, CAT, POD activity (**D**–**F**) under Cd treatment. Values are the means ± standard deviation (SD) (*n* = 3). Different letters in the same column indicate statistically significant differences (*p* < 0.05).

After treatment with 5 or 10 mg·kg^−1^ CdCl_2_, the H_2_O_2_, O_2_^•−^, and MDA contents increased significantly in wheat leaves, but their Cd-induced production, especially Cd-induced O_2_^•−^ production, was reduced by AMF (Figure 3A–C). The O_2_^•−^ production was reduced by 23.9%, 32.9%, and 28.4% in the inoculation group under 0, 5, and 10 mg·kg^−1^ CdCl_2_ treatments, respectively, compared to the non-inoculation group (Figure 3B). The SOD activity in the AMF inoculation group was significantly higher than that under normal conditions, but there was no significant difference between them under mg·kg^−1^ CdCl_2_ treatment. Even under 10 mg·kg^−1^ CdCl_2_ treatment, the SOD activity in the AMF symbiotic group was slightly lower than that in the non-symbiotic group (Figure 3D). The CAT activity was slightly enhanced by AMF symbiosis under non-Cd and Cd treatment (Figure 3E), and the change of POD activity was similar to CAT (Figure 3F). These results suggest that the alleviative effect of AMF on the oxidative damage induced by Cd may not mainly depend on the increase of SOD, CAT, and POD activity.

### 3.5. AMF Promotes the AsA-GSH Cycle in Wheat Leaves under Cd Stress

**Figure 4:**
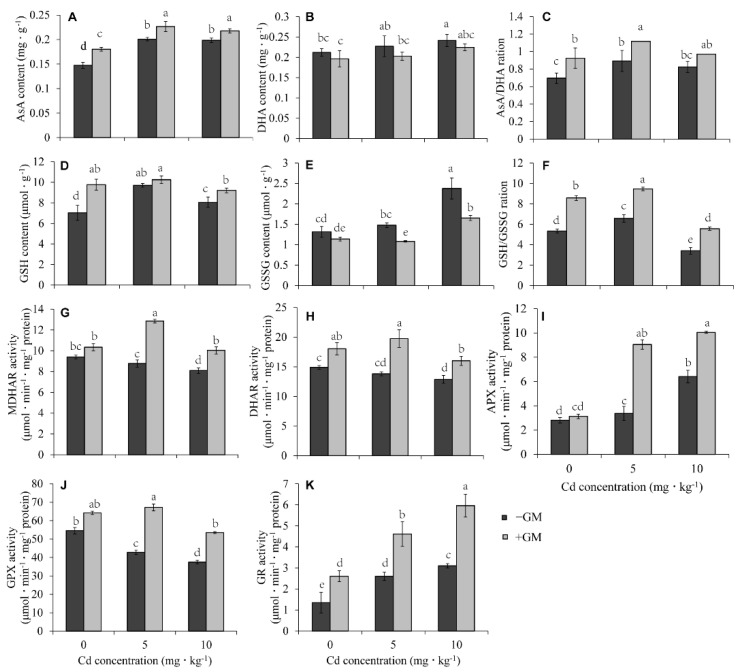
Effects of AMF on AsA-GSH cycle under different Cd concentrations. The content of AsA (**A**), DHA (**B**), GSH (**D**) and GSSG (**E**), the ratio of AsA/DHA (**C**) and GSH/GSSG (**F**), and the activities of MDHAR (**G**), DHAR (**H**), APX (**I**), GPX (**J**), and GR (**K**). Values are the means ± standard deviation (SD) (*n* = 3). Different letters in the same column indicate statistically significant differences (*p* < 0.05).

The AsA-GSH cycle system in plants is also an important ROS-scavenging mechanism. The redox status of AsA and GSH showed that AMF increased AsA and GSH contents but inhibited DHA and GSSG accumulation in the leaves under both non-Cd and Cd treatments (Figure 4A,B,D,E). The ratio of AsA/DHA and GSH/GSSG was significantly higher in the inoculation group than that in the non-inoculation group. The AsA/DHA ratios were 33.0%, 25.2%, and 17.8% higher in the inoculation group than in the non-inoculation group under 0, 5, and 10 mg·kg^−1^ CdCl_2_ treatments, respectively (Figure 4C). Similarly, the GSH/GSSG ratios were 60.6%, 43.9%, and 63.6% higher in the inoculation group than in the non-inoculation group (Figure 4F).

Several antioxidant enzymes are involved in the AsA-GSH cycle, and the analysis of their activities showed that AMF significantly increased the activities of MDHAR, DHAR, APX, GPX, and GR under both normal and Cd stress conditions compared to the non-inoculation group (Figure 4G–K). AMF increased the MDHAR activity by 10.0%, 46.5%, and 23.9%, DHAR activity by 21.1%, 43.0%, and 24.4%, APX activity by 11.2%, 167.8%, and 56.9% under 0, 5, and 10 mg·kg^−1^ CdCl_2_ treatments, respectively. The GPX activity was also increased by 17.8%, 56.8%, and 42.8%, while that of GR increased by 93.4%, 77.3%, and 92.1%, under 0, 5, and 10 mg·kg^−1^ CdCl_2_ treatments, respectively, upon the addition of AMF. Further, RT-qPCR analysis of the transcriptional level of these enzymes also showed that AMF could significantly induce their expression under Cd stress (Supplementary Materials S1). These results showed that AMF can accelerate the AsA-GSH cycle by increasing the activity of these enzymes.

### 3.6. AMF Promotes Methylglyoxal Degradation in Wheat Leaves under Cd Stress

**Figure 5:**
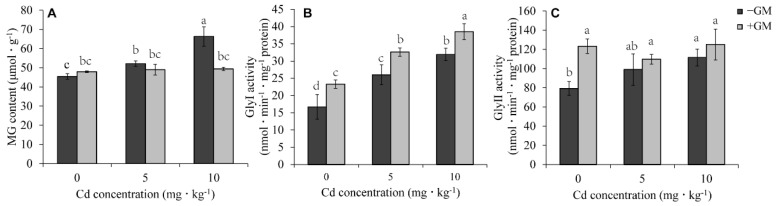
Effects of AMF on MG content (**A**) and its degradation-related enzyme Gly I and Gly II activities (**B**,**C**) under different Cd concentrations. Values are the means ± standard deviation (SD) (*n* = 3). Different letters in the same column indicate statistically significant differences (*p* < 0.05).

Methylglyoxal (MG), one of the toxins produced during glycolysis and protein and lipid peroxidation, can interfere with the physiological metabolism of plants by indirectly inducing oxidative stress, which further increases ROS production [56,57]. The toxic effects of MG in plants can be reduced through its degradation by Glyoxalase I (Gly I) and Glyoxalase II (Gly II) [58]. In the present study, Cd stress increased the MG content, which was significantly reduced by AMF, especially under a high concentration of Cd treatment (10 mg·kg^−1^ CdCl_2_), where the MG content in the inoculation group decreased by 25.5% compared with the non-inoculation group (Figure 5A). This reduction was due to AMF-induced activities of Gly I and Gly II. AMF increased Gly I activity by 39.5%, 25.4%, and 20.7% at 0, 5, and 10 mg·kg^−1^ CdCl_2_, respectively (Figure 5B). Although the AMF-induced activity of Gly II was not significant under Cd stress, the activity increased by 55.4% under non-Cd conditions (Figure 5C).

### 3.7. Effects of AMF on Phytochelatins and Metallothionein Contents of Wheat Leaves under Cd Stress

**Figure 6:**
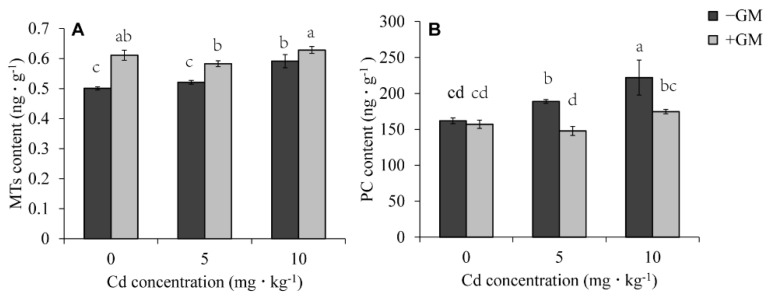
Effects of AMF on MTs (**A**) and PCs (**B**) content under different Cd concentrations. Values are the means ± standard deviation (SD) (*n* = 3). Different letters in the same column indicate statistically significant differences (*p* < 0.05).

Phytochelatins (PCs) and metallothionein (MTs) bind to HM ions to form chelates, which are then transported to the vacuole for detoxification [59]. Our results showed that AMF promoted the accumulation of MTs under both non-Cd and Cd treatments. AMF increased MT content by 22.0%, 11.9%, and 6.3% at 0, 5, and 10 mg·kg^−1^ CdCl_2_, respectively (Figure 6A). However, the content of PCs was significantly lower in the inoculation group than in the non-inoculation group (Figure 6B). These results implied that the detoxification of Cd in wheat leaves by AMF may mainly depend on promoting the chelation of Cd with MTs rather than PCs.

### 3.8. Analysis of Differentially Expressed Genes between the Inoculation and Non-Inoculation Groups

**Figure 7:**
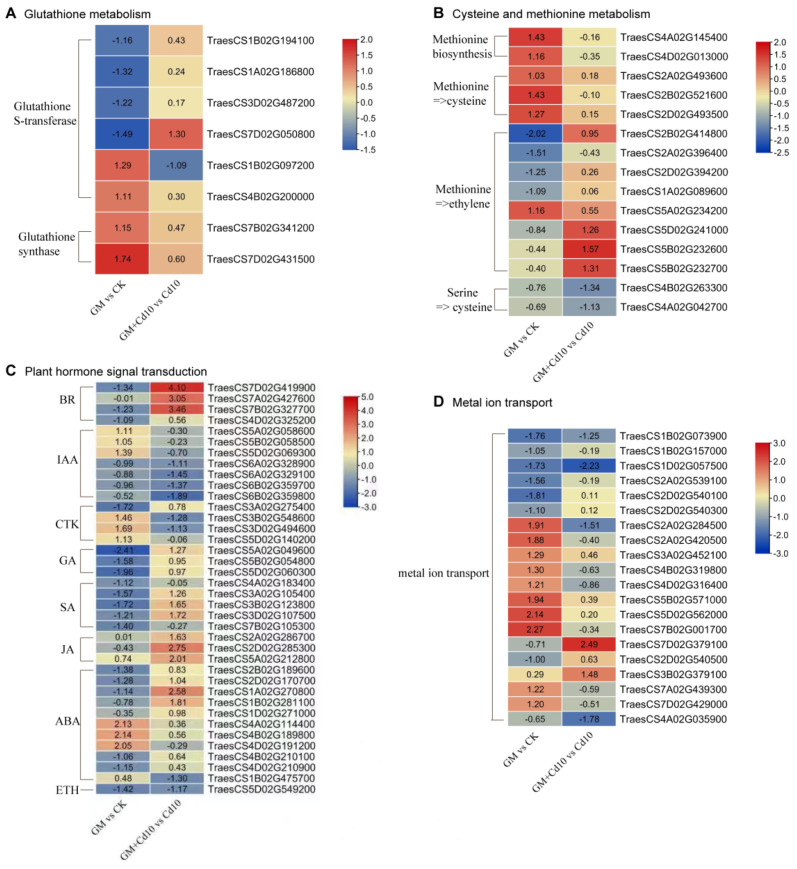
The DEGs mediating Glutathione metabolism (**A**), Cysteine and methionine metabolism (**B**), Plant hormone signal transduction (**C**) and Metal ion transport (**D**) in GM vs CK or GM+Cd10 vs Cd10 comparison groups. CK: Control group; GM: Inoculated with *Glomus mosseae* under normal conditions; Cd10: 10 mg·kg^−1^ CdCl_2_ treatment; GM+Cd10: Inoculated with *Glomus mosseae* under 10 mg·kg^−1^ CdCl_2_ treatment.

We compared the transcriptome changes in the four groups (CK, GM, Cd10, and GM+Cd10) to gain more insight into the molecular mechanism by which AMF alleviates Cd stress in wheat. Compared to the non-inoculation group, the inoculation group had 1826 (669 up-regulated and 1157 down-regulated) and 654 (338 up-regulated and 316 down-regulated) differentially expressed genes (DEG) under normal and 10 mg·kg^−1^ CdCl_2_ treatments, respectively (Supplementary Materials S2). The GO and KEGG enrichment analysis showed that the up-regulated DEGs in the GM vs. CK comparison group were significantly enriched in ‘metal ion transport’, ‘glutathione metabolism’, ‘cysteine and methionine metabolism’, and ‘plant hormone signal transduction’ terms (Supplementary Materials S2). These terms are closely related to the responses against heavy metal stress, suggesting that the Cd stress mitigation by AMF may be related to the genes found in these terms. Therefore, we screened for the DEGs associated with these four terms in the GM vs. CK or GM+Cd10 vs. Cd10 comparisons and found that 8, 15, 38, and 20 genes were involved in ‘glutathione metabolism’, ‘cysteine and methionine metabolism’, ‘plant hormone signal transduction’, and ‘metal ion transport’, respectively (Figure 7).

The genes involved in ‘glutathione metabolism’ were mainly GST and GR-related, and the expression of most GST genes decreased in the inoculation group compared with the non-inoculation group under the non-Cd treatment but increased under Cd treatment. The AMF symbiosis induced the expression of GS genes under both non-Cd and Cd stress (Figure 7A). These results suggest that the AMF-induced Cd stress tolerance is related to the GSH metabolic pathway regulated by the GST and GR genes.

The genes in ‘cysteine and methionine metabolism’ were mainly involved in methionine biosynthesis and the conversion of methionine to cysteine, serine to cysteine, and methionine to ethylene (Figure 7B). Under non-Cd conditions, AMF significantly induced methionine synthesis-related genes *TraesCS4A02G145400* and *TraesCS4D02G013000* (tyrosine aminotransferase and 5-methyltetrahydropteroyltriglutamate–homocysteine methyltransferase) and adenosylhomocysteinase (related to methionine-to-cysteine conversion) *TraesCS2A02G493600*, *TraesCS2B02G521600*, and *TraesCS2D02G493500*. However, the expression of serine O-acetyltransferase (*TraesCS4B02G263300* and *TraesCS4A02G042700*) involved in serine-to-cysteine conversion was decreased by AMF under Cd stress. Among the genes involved in the conversion of methionine to ethylene, 1-aminocyclopropane-1-carboxylate synthase *ACS* (*TraesCS2B02G414800*, *TraesCS2A02G396400*, and *TraesCS2D02G394200*) was inhibited by AMF under non-Cd conditions. However, the expression of aminocyclopropanecarboxylate oxidase *ACO* (*TraesCS5D02G241000*, *TraesCS5B02G232600*, and *TraesCS5B02G232700*) was induced by AMF under Cd conditions. These expression changes implied that AMF might affect methionine, cysteine, and ethylene levels under non-Cd and Cd treatment.

Previous studies showed that plant hormones mediate Cd stress responses. Similarly, we found that the Cd stress regulation by AMF was also mediated by genes related to plant hormone signal transduction (Figure 7C). After AMF inoculation, the expression of some genes involved in plant hormone signal transduction was repressed under non-Cd conditions but induced under Cd stress. These genes included xyloglucosyl transferase *TCH4* (*TraesCS7B02G327700*, *TraesCS7B02G327700* and *TraesCS7D02G419900*) and *BSK* (*TraesCS4D02G325200*) of the BR signaling pathway, and the phytochrome-interacting factor 4, *PIF4* (*TraesCS5A02G049600*, *TraesCS5B02G054800*, and *TraesCS5D02G060300*) of the GA signaling pathway. Others were protein *NPR1* (*TraesCS3A02G105400*, *TraesCS3B02G123800*, and *TraesCS3D02G107500*) of the SA signaling pathway, and the serine/threonine-protein kinase *SnRK2* (*TraesCS2B02G189600*, *TraesCS2D02G170700*, *TraesCS1A02G270800*, *TraesCS1B02G281100*, and *TraesCS1D02G271000*) of the ABA signaling pathway. Contrarily, AMF promoted the expression of a two-component response regulator, *ARR-A* (*TraesCS3B02G548600* and *TraesCS3D02G494600*), in the CTK signaling pathway under non-Cd conditions but inhibited its expression under Cd stress. Additionally, ethylene-responsive transcription factor 1, *ERF1* (*TraesCS5D02G549200*), was repressed by AMF under both non-Cd and Cd treatments.

The ‘metal ion transporters’ transport metals between different plant tissues, cells, or organelles. In this study, the AMF down-regulated 7 and up-regulated 10 metal ion transport-related genes significantly under non-Cd conditions, but down-regulated 4 and up-regulated 2 metal ion transport-related genes under Cd conditions (Figure 7D). Particularly, *TraesCS1B02G073900* and *TraesCS1D02G057500* were significantly repressed by AMF under both non-Cd and Cd conditions, and *TraesCS7D02G379100* and *TraesCS3B02G379100* were significantly induced by AMF under Cd stress. This suggested that these genes may be considered as important factors involved in the detoxification of cadmium by AMF.

**Figure 8:**
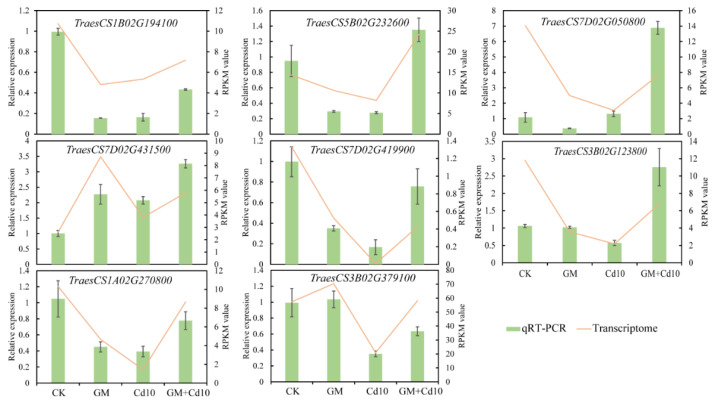
RT-qPCR analysis of eight representative genes in the metabolic pathways of ‘glutathione metabolism’, ‘cysteine and methionine metabolism’, ‘plant hormone signal transduction’, and ‘metal ion transport’.

Further, we selected eight representative genes for RT-qPCR analysis, including *TraesCS1B02G194100*, *TraesCS7D02G050800,* and *TraesCS7D02G431500* genes in the pathway of glutathione metabolism, *TraesCS5B02G232600* in cysteine and methionine metabolism, *TraesCS7D02G419900*, *TraesCS3B02G123800*, and *TraesCS1A02G270800* in plant hormone signal transduction, and *TraesCS3B02G379100* in the metal ion transporter pathway. The results showed that the RT-qPCR data were basically consistent with the changing trend of gene expression in the transcriptome (Figure 8).

### 3.9. Effects of AMF on Cysteine Content and Activities of Glutathione Synthetase and Glutathione S-Transferase

**Figure 9:**
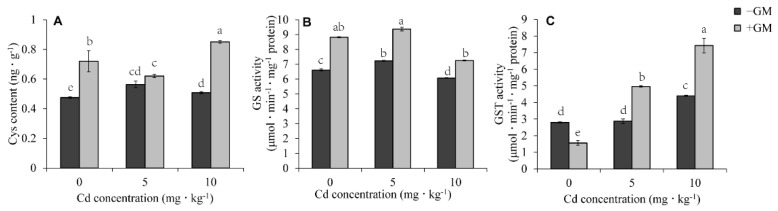
Effects of AMF on Cys content (**A**) and GS and GST activity (**B**,**C**) under different Cd concentrations. Values are the means ± standard deviation (SD) (*n* = 3). Different letters in the same column indicate statistically significant differences (*p* < 0.05).

Transcriptome data showed that AMF could significantly affect the expression of GS and GST genes in the ‘glutathione metabolism pathway’ and the expression of the genes related to the ‘cysteine and methionine pathway’. In order to verify the transcriptome data and clarify the comprehensive effect of these genes, we detected the cysteine content and the activity of GS and GST (Figure 9). As expected, AMF significantly promoted the Cys synthesis; for example, the content of Cys in the inoculation group increased by 51.2%, 10.2%, and 67.4% in the inoculation group under the treatments of 0, 5, and 10 mg·kg^−1^ CdCl_2_, respectively, compared with the non-inoculation group (Figure 9A). Compared to the non-symbiotic group, AMF symbiosis increased the GS activity 33.4%, 29.5%, and 19.4% under 0, 5, and 10 mg·kg^−1^ CdCl_2_, respectively (Figure 9B). The GST activity increased by 25.6% and 46.3% in the inoculation group under 5 and 10 mg·kg^−1^ CdCl_2_, respectively (Figure 9C).

## 4. Discussion

### 4.1. AMF Immobilizes Cd in Roots by Secreting GRPS and Reducing Cd Transport to Shoots

The mitigation effect of AMF on Cd stress has been demonstrated in several species, including *Medicago truncatula* [60], *Zea mays* [61,62], *Solanum photeinocarpum* [63], *Lonicera japonica* [64], and *Oryza sativa* [65]. We found that AMF reduced the inhibitory effect of Cd on wheat growth (Figure 1A–C). Moreover, previous studies have shown that AMF effects on Cd accumulation in plant tissues vary by plant species. According to Sterckeman [66], AMF may reduce, increase, or may not affect Cd accumulation in plant roots and shoots under different Cd treatments. However, the AMF-induced Cd increment in the roots and Cd decrease in the shoots was the main trend, accounting for 42% and 50%, respectively. Our findings showed that AMF significantly reduced Cd content in wheat shoots but increased it in roots (Figure 1D,E); that is, our results were consistent with the effects of AMF on Cd accumulation in most plants.

Glomalin, a glycoprotein secreted by AMF, has metal-binding properties, which could reduce the bioavailability of metals in plants [26]. Therefore, AMF alleviates HM stress by releasing glomalin to immobilize HMs in the rhizosphere [22,67]. Another study also reported that AMF significantly reduced Cd, Pb, Zn, and Cu concentrations in maize shoots by releasing glomalin (total-GRSP) [68]. Moreover, mycorrhizal fungi also increased the glomalin content and reduced the translocation of Cd in sorghum roots [55]. In this study, AMF significantly increased GRSP release in soil and the content of GRSP-bound Cd (Figure 2) but reduced Cd transportation from roots to shoots (Figure 1F). These results demonstrate that the AMF-secreted GRSP reduced the Cd content in shoots and alleviated cadmium stress in wheat.

### 4.2. AMF Promotes the ASA-GSH Cycle and MG Detoxification to Reduce ROS Production in Wheat Leaves

The accumulation of Cd in plants could induce active oxygen and hydroxyl radicals [69], resulting in leaf chlorosis, reduced photosynthesis and stomatal density, decreased mineral uptake, and reduced root nodulation [7]. Plants usually reduce oxidative damage by enhancing their antioxidant systems. For example, the SOD, APX, GR, MDHAR, and DHAR activities in Cd-tolerant mung bean (cv. Pusa 9531) were significantly higher than that in Cd-susceptible varieties (cv. PS 16) under Cd stress. The associated ration of AsA/DHA and GSH/GSSH was also much higher in cv. Pusa 9531 than that in cv. PS 16 [70]. Other studies have also reported that the alleviation effect of AMF on Cd-induced oxidative stress is also related to these antioxidant enzymes [22]. *Glomus versiforme* symbiosis improved the activities of CAT, POD, and APX in the leaves of *Solanum photeinocarpum* under Cd treatment, thus reducing lipid peroxidation [62]. Moreover, *Glomus versiforme* (Gv) and *Rhizophagus intraradices* (Ri) significantly increased CAT, APX, and GR activities in *Lonicera japonica* leaves [63]. In this study, we also found that AMF reduced the Cd-induced ROS production and lipid peroxidation in wheat leaves (Figure 3A–C); however, CAT and POD activities were just slightly higher in the AMF inoculation group than in the non-inoculation group, and AMF symbiosis did not significantly change the SOD activity, which was even lower than that in the non-symbiotic group under Cd stress (Figure 3D–F). On the other hand, the activity and transcription level of the antioxidant enzymes (MDHAR, DHAR, GR, and GPX) and antioxidant content (GSH and ASA) involved in the ASA-GSH cycle were remarkably higher in the inoculation group than in the non-inoculation group (Figure 4). Thus, we speculate that the inhibition of Cd-induced oxidative damage by AMF mainly depends on the acceleration of the ASA-GSH cycle. AMF also was found to reduce arsenic (As) toxicity in wheat leaves by promoting the ASA-GSH cycle [71].

MG is an organic compound that is toxic to plants since it disrupts the functions of biochemical-related reactions, including antioxidant enzymes, thereby indirectly increasing ROS production [58,72]. Moreover, MG can also function as a Hill oxidant and catalyze the photoreduction of O_2_ to O_2_^•−^ in photosystem I [73], and its detoxification is an efficient strategy for reducing ROS oxidative damage to improve plant abiotic stress tolerance. MG detoxification is the process of degrading MG into D-lactate by Gly I (Glyoxalase I) and Gly II (Glyoxalase I) with the help of GSH [58]. The AMF significantly increased Gly I activity (Figure 5B) and decreased the MG content under Cd treatment (Figure 5A), indicating that AMF could reduce oxidative damage by promoting MG detoxification. Previous studies have also found that AMF can alleviate As toxicity by increasing the activities of Gly I and Gly II [71]. In this study, AMF significantly reduced MG content and Gly I activity under Cd stress, which may weaken the stimulation of oxidative stress on Gly II, so we did not detect the significant induction of AMF on Gly II activity when exposed to Cd (Figure 5C).

### 4.3. AMF Promotes Cd Chelation or Sequestration

PCs and MTs are cysteine-rich, metal-binding polypeptides with excellent abilities for chelating heavy metals in plants. Both PCs and MTs can bind the HMs and sequester them into the vacuoles for detoxification [74]. For PC molecules, the longer the chain, the stronger the pH stability and the binding ability with heavy metals. Among the HMs, Cd is the most potent inducer of PCs synthesis in plants, for which the ratio of Cd to cysteine is about 2:1 in the structural model of a PC-Cd complex [75]. Many studies have shown that a lack of PC synthesis increases Cd sensitivity [13], but some have reported that PC accumulation has no positive effects on the enhancement of plant Cd tolerance. For example, the rice variety overexpressing the wheat *TaPCS1* gene was more sensitive to Cd than was the wild type [76]. Therefore, we speculate that the inhibitory effect of AMF on Cd-induced PC accumulation (Figure 6B) may also be a strategy to reduce Cd sensitivity, as shown by the low Cd content-induced PC synthesis in the symbiotic group. The same phenomenon was also observed in AMF-treated *Canavalia ensiformis* leaves, which showed reduced PC production at lower Cu levels [77].

MTs can bind different metal ions via cysteine thiol groups or sulfur molecules to aid their detoxification [78]. For example, heterologous expression of the metallothionein gene *PaMT3-1* in tobacco significantly enhanced tobacco tolerance to Cd [79]. Similarly, overexpression of *PpMT2* in *Arabidopsis* improved its tolerance to high concentrations of CdCl_2_ [80]. Expression of *OsMT1e* in a yeast Cd-sensitive strain *ycf1* conferred cellular tolerance to Cd, even though the *ycf1* + OsMT1e cells accumulated more Cd than the control cells [81]. Wheat Type 4 or EC (for early-cysteine labelled) MT is the only pMT for which 3D structural information is available, which contains 17 cysteines and can form two metal binding domains. Six cysteines in domain 1 can accommodate two Zn(II) or Cd(II) ions to form a binuclear M(II)2Cys6 cluster [82]. In our study, AMF significantly increased MT content in wheat leaves (Figure 6A), implying that MT detoxification of Cd is a mechanism by which AMF improves Cd tolerance in wheat.

In addition to PCs and MTs, GSH is also an effective heavy metal chelator. GSH can bind HMs to form GSH-HM complexes under the catalysis of GST, and the complexes are segregated into vacuoles for detoxification [83]. The role of GSH in heavy metal tolerance has been widely explored [84]. Exogenous GSH application alleviated Cd toxicity in wheat by regulating the absorption and translocation of Cd [85]. The GST decontamination effect on heavy metals has also been reported [86]; for example, overexpressing *OsGSTL2* in *Arabidopsis* significantly improved its tolerance to heavy metals [87]. Moreover, *GST* and *CYP2E1* co-expression in alfalfa aided the phytoremediation of mixed heavy metal-organic pollutants [88]. We found that AMF significantly increases GSH content (Figure 4D), glutathione synthase (GS) (Figure 9B), glutathione reductase (GR) (Figure 4K), GST activity (Figure 9C), and GST-related genes (Figure 7A) under Cd stress, suggesting that AMF alleviates Cd toxicity in wheat by promoting GSH synthesis and chelation Cd with the help of GST.

Heavy-metal-associated isoprenylated plant proteins (HIPPs) are a group of metal-binding metallochaperones that maintain heavy metal homeostasis, especially Cd tolerance, by enhancing their detoxification [89]. Overexpression of the *Arabidopsis* HIPPs gene, *CdI19*, increased plant Cd tolerance [90]. Additionally, transgenic rice overexpressing *OsHIPP42* displayed improved Cd resistance [91]. Both the *OsHIPP29* mutant and RNAi lines were sensitive to Cd [92]. *OsHIPP56* could repressed Cd toxicity by reducing its accumulation in rice [93]. In this study, two HIPPs family genes (*TraesCS7D02G379100* and *TraesCS3B02G379100*) were significantly induced by AMF under Cd stress, implying their involvement in the AMF-induced Cd detoxification.

### 4.4. Glutathione Metabolism Plays an Important Role in the Mitigation of Cd Stress in Wheat by AMF

GSH can be generated from the catalysis of γ-glutamylcysteine and glycine by glutathione synthase (GS), and through GSSG reduction by GR [83]. In this study, both the activity and transcriptional expression level of GS and GR were found to be significantly induced by AMF (Figure 4K, Figure 7A and Figure 8B), and at the same time, GSH accumulation was detected to be promoted by AMF under both non-Cd and Cd treatments (Figure 4D). This meant that the maintenance of GSH homeostasis by AMF under Cd stress was attributed to GS and GR.

GSH mitigates Cd stress by reacting directly with H_2_O_2_, O_2_^•−^, and OH· to form adducts or participate in the ASA-GSH cycle to scavenge ROS [94]. GSH can also bind HM ions under the catalysis of GST to form non- or less-toxic complexes transported to the vacuole for detoxification. Moreover, GSH is directly involved in the two-step detoxification process of MG [83]. Coincidentally, while AMF promoted the accumulation of GSH under Cd stress, the activities of GST (Figure 9C) and these antioxidant enzymes involved in the ASA-GSH cycle (Figure 4G–K) were induced by AMF, and the content of ROS (Figure 3) and MG (Figure 5A) was reduced by AMF. Thus, these results showed that GSH metabolism plays a pivotal role in mitigating Cd stress in wheat via AMF induction.

### 4.5. Plant Hormone Signaling Mediates the AMF Alleviation of Cd Stress in Wheat

Abscisic acid (ABA), ethylene (ETH), auxin, brassinosteroid (BR), and salicylic acid (SA) have been found to regulate plant responses to heavy metal stress [95,96,97,98]. We observed that AMF could regulate multiple genes related to ‘plant hormone signal transduction’ under both normal and Cd stress conditions (Figure 7C), indicating that AMF may also regulate Cd stress through the hormone signaling pathway.

ABA plays a vital role in alleviating heavy metal and metalloid stresses in plants, and the uptake and accumulation of toxic heavy metals in crops can be reduced via ABA signaling transduction [99]. ABA can limit the long-distance transfer of HMs from root to shoot by closing stomata and reducing transpiration rate. It also can reduce the effects of HM stress and promote plant growth by improving the antioxidant defense system, osmolytes, and metal chelates [100]. The *Arabidopsis* ABA-insensitive double mutant *snrk2.2/2.3* abolished exogenous ABA-induced reduction in Cd accumulation compared to WT [101]. In the present study, five *SnRK2* genes (*TraesCS2B02G189600*, *TraesCS2D02G170700*, *TraesCS1A02G270800*, *TraesCS1B02G281100*, and *TraesCS1D02G271000*) were found to be induced by AMF under Cd stress (Figure 7C), and the Cd accumulation in shoots of the AMF symbiotic group was lower than that of the non-symbiotic group under Cd stress (Figure 1D). These results suggest that AMF may mediate ABA signaling by inducing *SnRK2* expression, thereby reducing Cd accumulation and its associated oxidative damage in wheat leaves.

Ethylene also plays a pivotal role in HM stress tolerance [102]. HM stress enhances ethylene production by inducing the two key enzymes, ACS and ACO, involved in ethylene synthesis [103,104]. Ethylene is synthesized from methionine, which is converted to S-adenosylmethionine (SAM) by SAM synthetase, and then SAM forms ACC (1-aminocyclopropane-1-carboxylic acid) under the action of ACS; subsequently, ACC is oxidized into ethylene by ACO [102]. From the transcriptome results, we observed that AMF significantly up-regulates the expression of three *ACO* genes (*TraesCS5D02G241000*, *TraesCS5B02G232600*, and *TraesCS5B02G232700*) under Cd stress (Figure 7B), indicating that AMF might promote the conversion of methionine to ethylene to regulate Cd stress. Conversely, AMF inhibited the expression of ACS (*TraesCS2B02G414800*, *TraesCS2A02G396400*, and *TraesCS2D02G394200*) under normal conditions, but up-regulated adenosylhomocysteinase (*TraesCS2A02G493600*, *TraesCS2B02G521600*, *TraesCS2D02G493500*) involved in the methionine→cysteine pathway. Coincidentally, the cysteine content of the AMF symbiotic group was significantly higher than that in the control group (Figure 9A). Therefore, these results suggest that methionine was more converted to cysteine than ethylene under normal conditions. Cys provides a substrate for the synthesis of GSH and is an important component of the conservative motif in MTs. Naturally, the accumulation of Cys is conducive to GSH and MT production. In fact, we did find that AMF can increase GSH and MTs under Cd stress (Figure 4D and Figure 6A). Undoubtedly, these changes promoted the alleviative effects of AMF on Cd stress toxicity.

BR is also recognized as an important hormone regulating plant tolerance to heavy metals [105]. BR overcomes HMs’ toxicity mainly through enhancing antioxidant capacity, reducing the absorption of HMs, and improving plant growth. Studies showed that the promoting effects of BR on the growth of shoot and root is mainly the result of cell elongation and expansion [106]. In this study, *TCH4* (*TraesCS7B02G327700*, *TraesCS7B02G327700*, and *TraesCS7D02G419900*), which mediates the BR signal and is responsible for cell elongation, was found to be significantly induced by AMF under Cd stress; moreover, AMF also slightly induced *BSK* (*TraesCS4D02G325200*) under Cd stress (Figure 7C). This suggested that AMF may promote wheat growth through a BR signaling pathway to resist Cd stress.

SA could relieve HM stress in plants by inducing antioxidant compounds and enzymes [98]. *NPR1*, an SA receptor, is a linker between SA signaling molecules and the defense gene activation [107]. Our study found that *NPR1* (*TraesCS3A02G105400*, *TraesCS3B02G123800*, and *TraesCS3D02G107500*) was significantly up-regulated by AMF under Cd stress, indicating that the alleviating of Cd-induced oxidative damage by AMF may be associated with its mediation of the SA signaling pathway.

## 5. Conclusions

**Figure 10:**
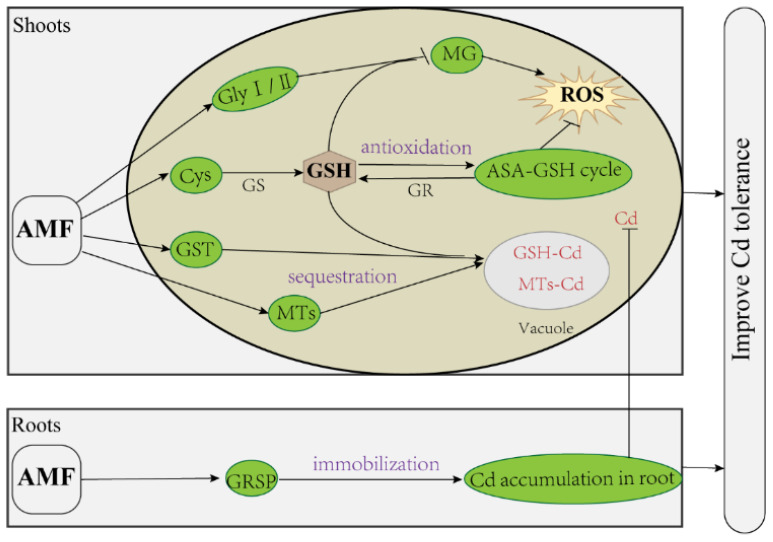
The proposed regulatory mechanism model of AMF to alleviate Cd stress in wheat. Arrow and bar ends indicate activation and inhibitory effects, respectively. AMF: arbuscular mycorrhizal fungi; Gly I/II glyoxalase I/II; MG: methylglyoxal; Cys: cysteine; GS: glutathione synthase; GSH: glutathione; GR: glutathione reductase; AsA-GSH cycle: ascorbate-glutathione cycle; GST: glutathione S-transferase; MTs: metallothionein; ROS: reactive oxygen species; GRPS: glomalin related soil protein.

AMF symbiosis is an inexpensive and eco-friendly strategy for bioremediation. In this study, AMF alleviated Cd stress in wheat. After determining the Cd, GRPS, ROS, ASA, GSH, Cys, MTs, PCs, and MG contents, the activities of the associated antioxidant enzymes, and the transcriptome, we propose that three regulatory mechanisms are involved in Cd stress alleviation by AMF in wheat (Figure 10). Firstly, AMF immobilizes Cd in the roots by secreting GRPS, thus decreasing the translocation of Cd to the shoots. Secondly, AMF reduces Cd-induced ROS production and oxidative damage by accelerating MG degradation and the AsA-GSH cycle. Finally, AMF promotes Cd chelation and sequestration by inducing the production of GSH and MTs. Additionally, GSH plays an important role in both anti-oxidation and Cd chelation; therefore, we consider that the regulation of GSH homeostasis by AMF may be its key pathway to alleviate Cd stress in wheat.

## Data Availability

Data is contained within the article.

## Supplementary Materials

The following supporting information can be downloaded at: https://www.mdpi.com/article/10.3390/antiox11122390/s1. Supplementary materials S1: Root colonization of wheat inoculated with GM; Transcriptional expression of related enzymes in AsA-GSH cycle under Cd stress; Primers used in this study. Supplementary materials S2: The information of differentially expressed genes (DEG) under normal and 10 mg·kg^−1^ CdCl_2_ treatments.
