# Supplementary material for: Physio-Biochemical and Transcriptomic Features of Arbuscular Mycorrhizal Fungi Relieving Cadmium Stress in Wheat
Source: Antioxidants (Basel). 2022 Dec 1;11(12):2390. doi: 10.3390/antiox11122390 (PMC9774571; doi:10.3390/antiox11122390)
Supplement: Supplementary file 1 [file antioxidants-11-02390-s001.zip › Supplementary data/Supplementary materials S1.pdf]

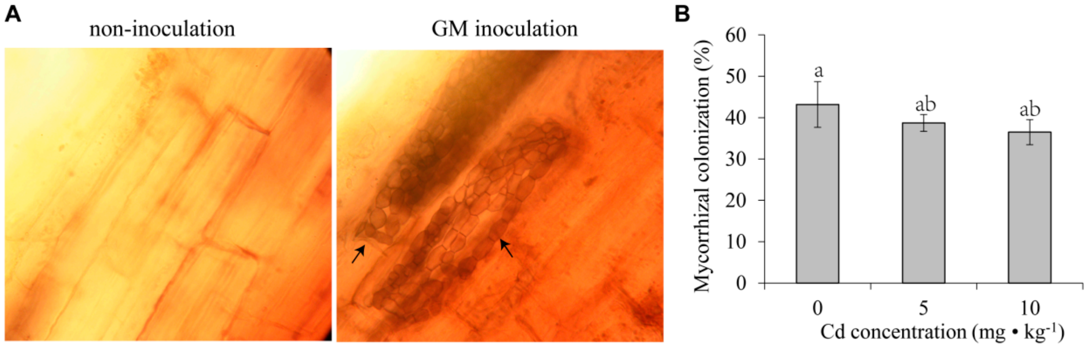

Figure. S1 Root colonization of wheat inoculated with *Glomus mosseae* (GM). (A) Images of the ink-vinegar solution staining of the intraradical hyphae (where the arrow points). (B) Root colonization rates (%) of wheat inoculated with GM under 0, 5 and 10 mg·kg^{-1} $CdCl_2$. Different letters indicate significant differences under different Cd treatments at the level of $P < 0.05$.

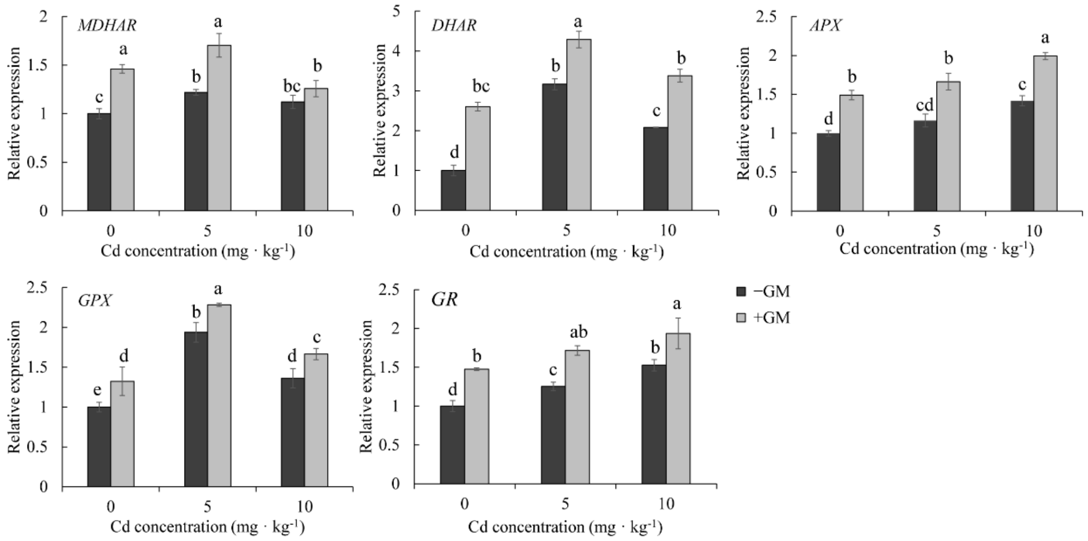

Figure. S2 Transcriptional expression of related enzymes in AsA-GSH cycle under Cd stress.

Table S1 Primers used in this study.

Gene name	Primer sequences	
	Forward (5'-3')	Reverse (5'-3')
MDHAR	CTTACAGCCACAGGAGAAACTA	CAGCATCTGAACCGCTTATT
DHAR	GGTGCCCTACCAGATGAA	CGTGACAAAGGTGGAGAAG
APX	CATTACCACCGACCTTCAGGC	CTACGCTGAGGCACACCTCAAG
GPX	CTCCGTCCATGACTTCGT	CCAATCCTTTCTCCCTGT
GR	GCACACGACCAAGCACATAT	ATATCCGCCACCAAGAATAACG
TraesCS1B02G194100	CTGGGTAGGCATCATGTGGG	CCTCCAATTGGCCGATAGCC
TraesCS7D02G050800	GACGGTGGAGATGAAGGTCC	TTCTCCCTGGGGACAACGTC
TraesCS7D02G431500	AACGCATTTACCAGCGCTTC	CAAGCACGACGAAGACAACG
TraesCS5B02G232600	CTGGGAGAGCACCTTCTTCG	CCAGCTTCTCCAGCTCTGAC
TraesCS7D02G419900	GTGGCGATAGGCATTCTGG	GAGGTCTTGTCGAGCGTGAG
TraesCS3B02G123800	ATGAACCTGAGCTGGCTTCC	TCTCCTAGTTCGACCTGCCA
TraesCS1A02G270800	GATCTGCGGCGTAAGCTACT	ATCGCATATCTTGAGCCGGG
TraesCS3B02G379100	ATGGATGGATGCTGGCAGG	CCGATCTTCTGCAGAGCCTT
Actin	ACAGAGAGAAAATGACCCA	AGGATAGCATGAGGAAGC
